# A Double-Blind Randomized Active-Controlled Trial Evaluating the Short-Term Efficacy of a Single Intramuscular Injection of a Fixed-Dose Combination Product Containing Diclofenac and Thiocolchicoside in Patients with Acute Moderate to Severe Low Back Pain

**DOI:** 10.3390/jcm14248827

**Published:** 2025-12-13

**Authors:** Ioannis Oikonomou, Maria Panagiotopoulou, Nikolaos Christopoulos, Eleni Loukeri, Georgios Christodoulakis, Georgios Kountis, Stamatios A. Papadakis, Ioannis Chiotis, Athanasios Georgokostas, Nikolaos Garantziotis, Christos Loukas, Dimitrios Pallis, Petros Nikolakakos, Yiannis C. Bassiakos

**Affiliations:** 1 Department of Medicine, University of Patras, 265 04 Rio, Greece; 2 Verisfield Single Member S.A., 152 31 Halandri, Greece; maria-pan@outlook.com (M.P.); nchristop.ph@gmail.com (N.C.); el_louk@windowslive.com (E.L.); 3 Orthopaedic Department, Peiraiko Therapeftirio S.A., 185 34 Piraeus, Greece; christodoulakisgeorge@yahoo.gr (G.C.); info@chiotisioannis.gr (I.C.); georgokostasortho@gmail.com (A.G.); 4 5th Orthopaedic Department, KAT General Hospital, 145 61 Kifissia, Greece; geokoundis@gmail.com (G.K.); ngarantz.cod@gmail.com (N.G.); cloykas@gmail.com (C.L.); 5 2nd Orthopaedic Department, KAT General Hospital, 145 61 Kifisia, Greece; sanpapadakis@gmail.com (S.A.P.); dimitrispallis99@gmail.com (D.P.); petros_nikolakakos@hotmail.com (P.N.); 6 Department of Economic Sciences, National & Kapodistrian University of Athens, 105 59 Athens, Greece; ybassiak@econ.uoa.gr

**Keywords:** low back pain, diclofenac, thiocolchicoside, combination therapy, muscle spasm, randomized trial, acute pain

## Abstract

**Background/Objectives**: Acute low back pain (LBP) is a prevalent cause of disability worldwide. If often involves both inflammation and reflex muscle spasm, suggesting combined therapy with a non-steroidal anti-inflammatory drug (NSAID) and a muscle relaxant may provide superior relief. This study aimed to evaluate the short-term efficacy and safety of a single intramuscular (IM) injection of a fixed-dose combination (FDC) product containing Diclofenac and Thiocolchicoside versus Diclofenac monotherapy in adults with acute LBP. **Methods**: We conducted a phase III multicenter, randomized, double-blind, parallel-group trial in 140 patients with acute LBP of moderate to severe intensity. Patients were allocated 1:1 to receive either the combination of Diclofenac sodium 75 mg + Thiocolchicoside 4 mg (FDC product, Test Group) or Diclofenac sodium 75 mg alone (Diclofenac monotherapy, Reference Group) via a single IM injection. The primary outcome was the change in patient-reported pain intensity using the Visual Analogue Scale (VAS) from baseline to 3 h post-dose. Key secondary outcomes included pain change at 1 h in the VAS, improvement in lumbar mobility (finger-to-floor distance test, FTF), the proportion of patients achieving >30% pain reduction, and the incidence of adverse events (AEs). Randomization was centralized and both investigators and patients were blinded to the treatment. **Results**: All 140 randomized patients completed the trial. At 3 h post-injection, the combination therapy produced a significantly greater mean pain reduction than monotherapy (41.52 mm vs. 28.13 mm on the 100 mm VAS; *p* < 0.0001). Superiority of the combination was already evident at 1 h post-dose (VAS reduction 27.61 mm vs. 20.40 mm; *p* = 0.0089). Lumbar flexion improved more with the combination at 3 h (mean FTF distance improvement 14.52 cm vs. 7.94 cm; *p* < 0.0001) and at 1 h (9.21 cm vs. 4.62 cm; *p* < 0.0001). A higher proportion of patients achieved >30% pain relief with the combination (45.7% vs. 27.2% at 3 h, *p* < 0.0001; 31.4% vs. 18.6% at 1 h, *p* = 0.0066). Only one mild, transient adverse event (headache) was reported in the combination group (1.4% of patients) and none in the diclofenac-only group. No serious AEs occurred. **Conclusions**: A single intramuscular dose of diclofenac plus thiocolchicoside provided faster, greater relief of acute LBP than diclofenac alone, without compromising safety. The combination therapy significantly reduced pain and muscle spasm-related mobility limitations within 1–3 h and was well tolerated. These results support the combined use of an NSAID and muscle relaxant as an effective and safe option for acute moderate-to-severe LBP management.

## 1. Introduction

Acute LBP, defined as pain lasting less than four weeks [1,2], is an extremely common global health problem [3], consistently ranked as the leading cause of years lived with disability (YLDs) worldwide [3,4]. The lifetime prevalence of LBP across adults ranges broadly, affecting up to 84% of individuals [5,6]. In resource-rich countries, over 70% of people will experience LBP at some point in their lives [7]. More specifically focusing on acute episodes, the one-year prevalence of acute LBP is reported to be as high as 38% [8], and approximately one quarter of U.S. adults reported having LBP lasting at least one day in the past three months [1,2]. LBP is one of the most common reasons individuals seek consultation with general physicians in the developed world [8,9]. While acute LBP is generally self-limiting, with many episodes resolving on their own [1,6], recurrence rates are notably high, with symptoms recurring in 50% to 80% of people within one year of the initial episode [7]. Furthermore, despite initial rapid improvement, up to one third of patients may still report persistent back pain of at least moderate intensity one year following an acute episode [1], highlighting the profound personal and socioeconomic burden this condition imposes globally [1,2].

The pathophysiology of acute LBP is often challenging to define, with estimates suggesting that in up to 90% of cases, a specific pathoanatomical cause cannot be determined, leading to a classification of non-specific LBP [10,11]. Mechanically, LBP can originate from age-related degenerative processes or from musculo-ligamentous injuries [12]. Acute episodes may be triggered by exposure to physical factors such as bending, twisting, or lifting, as well as psychosocial elements like tiredness, anxiety, stress, or depression [13]. One of the most common clinical features of acute LBP is muscle spasm, which arises from the irritation of muscles, intervertebral discs, or ligaments [12]. The persistence of symptoms is often explained by the pain-spasm-pain cycle, a generally accepted theory [14]. This cycle posits that an initial insult or pain-evoking event leads to muscle spasm, which, by inducing pain and restricting range of motion, causes further muscle contractions [15,16]. This mechanism results in the accumulation of inflammatory mediators and the self-perpetuation of the cycle, potentially contributing to the chronicity of the LBP [15,16]. Crucially, this resultant muscle spasm significantly reduces mobility, severely disturbing daily function and physical activity [12].

The combination of diclofenac, an NSAID, and thiocolchicoside, a muscle relaxant, is used for acute LBP to target both inflammation and muscle spasm, offering a dual mechanism aimed at interrupting the pain-spasm cycle and achieving early symptomatic relief and improved mobility [17]. Several primary studies support the efficacy of this multimodal approach against NSAID monotherapy. A prospective study in patients with acute and subacute LBP found that combination therapy of Diclofenac sodium (75 mg twice daily) plus Thiocolchicoside (4 mg twice daily) for seven days was significantly more effective than Diclofenac sodium alone in decreasing pain scores (measured by VAS) and reducing disability (measured by hand-to-floor distance) by day 7 [12]. Furthermore, a randomized controlled single-blind trial investigating a single IM FDC of diclofenac (75 mg) and thiocolchicoside (4 mg) in acute moderate-to-severe LBP demonstrated its superiority over diclofenac monotherapy, providing rapid and sustained improvement in pain intensity and mobility within three hours post-injection. For instance, this single FDC administration resulted in a statistically significantly higher percentage of patients achieving a clinically meaningful reduction in pain intensity (>30%) at three hours compared to diclofenac alone [18]. An oral FDC of potassium diclofenac (50 mg) and thiocolchicoside (4 mg) also resulted in substantial pain reduction (over 85% on VAS) and near-complete resolution of muscle spasms compared to placebo in patients with acute lumbar pain of functional origin [19]. When comparing an FDC IM injection versus the separate injection of the two components, the FDC was found to be equally effective and well-tolerated over a five-day treatment period for acute moderate-to-severe LBP [16]. Tolerability data further reinforce its use, with some trials reporting the combination to be well-tolerated with no adverse drug reactions, whereas the monotherapy diclofenac group reported instances of mild dizziness [17].

A recent systematic review [17] concluded that the combination therapy of diclofenac and thiocolchicoside offers significant pain relief and functional improvement, particularly in acute LBP cases, owing to the synergistic effects of its anti-inflammatory and muscle-relaxant properties [17]. However, the overall certainty of evidence for pain reduction assessed using the VAS was judged to be low, primarily due to the high risk of bias across most of the included studies. Consequently, the combination may be considered a viable option for acute pain management when muscle spasm is a dominant feature, but further rigorous, standardized research is essential to definitively establish its efficacy and safety profile for clinical decision-making [17].

The present study was designed against the identified backdrops to provide a robust evaluation of the efficacy and safety of a fixed-dose combination of diclofenac and thiocolchicoside for acute LBP of moderate to severe intensity. We conducted a double-blind randomized active-controlled multicenter trial to confirm the superior pain-relieving efficacy of the combination, administered as a single IM injection, compared to diclofenac alone as measured by patient VAS scores.

## 2. Materials and Methods

### 2.1. Study Design

This was a phase III double-blind, active-controlled, multicenter clinical trial conducted in clinical sites in Greece. The trial had a two-arm parallel group design with 1:1 allocation ratio between the investigational FDC product and the active control. The study protocol was developed in accordance with the ICH E6 Good Clinical Practice (GCP) Guidelines, the European Medicines Agency’s (EMA) Guideline on Clinical Development of Fixed Combination Medicinal Products and ICH Topic E9 Statistical Principles for Clinical Trials. The trial uses the acronym DITH/VER. The protocol was registered in the Clinical Trials Information System (CTIS) in accordance with EU Clinical Trials Regulation No. 536/2014 (EUCT Number 2024-512786-13-00) and was approved by the Greek National Competent Authorities (National Organization for Medicines, EOF) and the Greek National Ethics Committee on 28 August 2024. The overall study design followed that of prior studies [18], but with important enhancements to reduce bias.

The treatment period was a single day, and each patient participated in four study visits. Visit 1 involved the informed consent processes, eligibility confirmation, baseline assessments and the administration of the investigational medicinal product (IMP). Visit 2 took place on-site at 1 h ± 15 min post-dose and involved efficacy and safety assessments. Visit 3 also occurred on-site at 3 h ± 15 min post-dose and involved the primary endpoint evaluation and safety assessment (Test of Cure Visit). Visit 4 was remote and was performed via phone call at 24 ± 4 h post-dose for safety monitoring. Unscheduled on-site visits were allowed at investigators’ discretion for any emergent safety concerns.

Given the need for rapid analgesia in acute LBP, this short, early-outcomes design was considered appropriate: IM diclofenac provides relief within ~15–30 min [20], and other studies [16,18] have shown meaningful improvements at 1 and 3 h for both an FDC and diclofenac alone. EU-authorized Summaries of Product Characteristics also support this regimen with diclofenac 75 mg IM products typically administered for a maximum of 2 daily doses for 2 days, and thiocolchicoside 4 mg IM products for a maximum of 2 daily doses for 5 days. Although diclofenac’s plasma half-life is ~1–2 h, sustained efficacy is expected due to high concentrations at inflamed sites (detectable in synovial fluid ~11 h; synovial half-life 3–6 h) and ongoing PGE_2_ inhibition [21,22,23,24,25,26]. A 24 h safety check was also considered appropriate because most IM-related adverse events occur early. Given thiocolchicoside’s aneuploidogenic risks, limiting exposure and focusing on early endpoints aligns with the “lowest effective dose, shortest duration” principle.

The study was multicenter to enhance generalizability, enrolling patients from three orthopedic departments in Greece, one of which belonged to a private clinic and the other two to a public hospital. All participating investigators were GCP-trained and have demonstrated experience in managing acute LBP and conducting clinical trials. All sites followed the same protocol and measurement procedures, with oversight by monitors to ensure protocol compliance and data quality. The possibility of activating additional sites existed to ensure the achievement of the required sample size within the planned timelines. To minimize potential selection bias, physicians were encouraged to consecutively enroll and document all subjects who met the site-specific targets and study eligibility criteria over the predetermined recruitment period. Before any study-related procedure was performed, each potential subject provided voluntary written informed consent.

No changes were made to the conduct of the study or to the planned statistical analyses after study initiation. All procedures, eligibility criteria, treatment regimens, sample size specifications, and analysis methods were implemented as originally described in the approved protocol and the Statistical Analysis Plan (SAP). No treatment arms were dropped, no entry criteria or drug dosages were modified, and no interim analyses or re-estimations of sample size were conducted. All study conduct and analytical approaches were executed without amendment prior to database lock and unblinding. Consequently, there are no changes that impact the interpretation of the study results. Patients and the public were not involved in the design, conduct, or reporting of this research.

The trial was conducted and reported in accordance with the CONSORT 2025 guidelines (see Supplementary Materials).

### 2.2. Participants

Subjects were eligible for inclusion if they were ≥18 years old; presented with acute LBP of ≤7 days’ duration with moderate to severe intensity (VAS ≥ 50 mm); were able to understand the clinical trial requirements and agree to attend the required follow-up visits; and were willing to provide voluntary written informed consent before any trial-related procedure.

Although one other study classified moderate–severe pain from ≥40 mm [18], the present study adopted ≥50 mm to better reflect the validated “moderate” range and the practice of comparable acute LBP studies [3,16,17]. A ≥50 mm VAS threshold is justified by multiple converging lines of evidence: studies mapping VAS to categorical pain consistently define mild as ~5–44 mm, moderate as ~45–74 mm, and severe as ~75–100 mm [27]. Therefore, 50 mm falls squarely in the moderate-or-higher range. Collins et al. showed “moderate” pain averages ~49 mm (SD ± 17) and “severe” ~75 mm (SD ± 18), with 85% of severe cases >54 mm, reinforcing 50 mm as a clean cut for at least moderate pain [28]. Word-anchoring experiments place “moderate” around ~44–45 mm (±13), again compatible with a 50 mm cutoff [29], and other postoperative studies similarly bracket 45–74 mm as moderate [30]. By contrast, a ≥40 mm rule risks enrolling patients with only mild pain. In acute LBP, where minimum important difference (MID) is ~10 mm [17,31] and minimum clinically important difference (MCID) is ~35 mm [32,33], starting at ≥50 mm ensures sufficient headroom to detect clinically meaningful improvement, yielding a clinically significant baseline and a robust test of treatment efficacy. This choice also preserves sufficient headroom on the VAS to register clinically important change if treatment is effective.

Subjects were excluded if their back pain was due to metastatic cancer, spinal infection, or spinal cord compression; if they had a history or presence of peptic ulceration, gastrointestinal bleeding, or severe dyspepsia; a history of inflammatory bowel disease (Crohn’s disease or ulcerative colitis); allergy to NSAIDs or skeletal muscle relaxants; asthma or other allergic disorders induced by acetylsalicylic acid or NSAIDs; use of NSAIDs or skeletal muscle relaxants within the 24 h prior to study entry; a history of concurrent systemic disease; a history of thrombopenia, easy bruising, haemophilia, or coagulation factor deficiency; established congestive heart failure (NYHA II–IV), ischemic heart disease, peripheral arterial disease, or cerebrovascular disease; hepatic or renal failure; poorly controlled arterial hypertension; treatment with ACE inhibitors for arterial hypertension; treatment with anticoagulants (vitamin K antagonists, new oral anticoagulants, or LMWH) or antiplatelet agents (glycoprotein platelet inhibitors such as abciximab, eptifibatide, or tirofiban, or platelet aggregation inhibitors such as aspirin, clopidogrel, dipyridamole, prasugrel, ticlopidine, or ticagrelor); pregnancy, breastfeeding, or childbearing potential without highly effective contraception; participation in another trial within the last 30 days using IMPs or a device; unwillingness or inability to comply with trial procedures or to consent to the storage, saving, and transmission of pseudonymous medical data; or legal incapacity or detention.

The selection of exclusion criteria for the DITH/VER study was founded on a comprehensive scientific and ethical rationale, aiming to prioritize participant safety, ensure the integrity and validity of the trial’s results, and establish a clinically relevant and homogeneous study population for evaluating acute moderate-to-severe LBP. These criteria are meticulously chosen based on the known pharmacological profiles of Diclofenac and Thiocolchicoside, the specific nature of low back pain, and general principles of GCP [6].

### 2.3. Interventions and Blinding

There were two IMPs in this study; the Test FDC product (Diclofenac sodium + Thiocolchicoside/Verisfield solution for injection (75 + 4) mg/ampule, Verisfield Single Member S.A., Halandri, Greece) and the Reference Diclofenac monotherapy product (Voltaren^®^, Diclofenac sodium solution for injection, 75 mg/ampule, Novartis (Hellas) AEBE, Metamorfosi, Greece).

Since the trial was conducted under a double-blind design, measures were in place to blind both participants and investigators assessing study outcomes to the treatment assignments. A challenge existed because the Reference product was a clear, colourless liquid, while the Test product was yellowish. A label sticker was affixed to the ampoules, but their content remained visible. To manage this limitation and preserve the blind, the study implemented a blinding strategy involving role separation. Each trial site assigned qualified unblinded personnel who were exclusively responsible for the preparation and administration of the IMPs. These individuals had no involvement in subject assessments, data collection, or any other trial procedures of the subjects to whom they had administered an IMP. Investigators and other study staff involved in outcome evaluation were blinded and prohibited from accessing information that could reveal treatment assignment. Similarly, the unblinded personnel were instructed not to disclose or imply any details regarding treatment identity to either subjects or blinded staff. Blinding integrity was assessed during the study monitoring visits, where study monitors evaluated the packaging and labeling integrity, as well as the location and accountability of physical records in each site, including the source documents. The integrity of the blinding was also preserved via the Electronic Data Capture (EDC) system as each assessing investigator only had access (via investigator-specific login credentials) to the patients they enrolled and assessed, and not to patients enrolled and assessed by other investigators, even if those operated within the same investigational site. The Reference product underwent secondary repackaging so that both Test and Reference products were presented in identical white carton boxes, each containing a single vial. The study monitors and patients were also blinded to the treatment assigned. Unblinding was permitted only in cases of emergency, such as when knowledge of the assigned treatment was necessary for subject safety. Unblinding procedures were incorporated into the EDC system.

### 2.4. Outcomes and Assessments

The primary endpoint was the mean change in patient-reported pain intensity from baseline (pre-dose) to 3 h post-dose, measured by a 100 mm VAS. The 3 h post-dose time point was designated as the “Test of Cure” visit, reflecting the expected peak effect of the medications.

Secondary endpoints included: (1) Mean pain intensity change from baseline to 1 h post-dose (Visit 2) on the VAS, to evaluate earlier onset of analgesia, (2) proportion of patients with >30% reduction in pain intensity from baseline at 3 h post-dose, (3) Proportion of patients with >30% reduction in pain intensity from baseline at 1 h post-dose, (4) Mean change in FTF distance from baseline to 1 h post-dose, and (5) Mean change in FTF distance from baseline to 3 h post-dose. Additional secondary safety endpoints were chosen relating to the proportion of patients withdrawn for reasons related to treatment and the proportion of patients experiencing adverse events (AEs).

The VAS was a horizontal line anchored with “no pain” (0) and “worst imaginable pain” (100); patients marked their current pain level, and the distance in millimeters from 0 was recorded as the score.

The FTF test is an objective physical measurement designed to quantify spinal/lumbar flexion or mobility. In this test, a patient stands and bends forward as far as possible without bending their knees, and the distance between their fingertips and the floor is measured. This provides a direct indicator of how low back pain impacts a patient’s range of motion, especially in movements involving forward bending.

Both the VAS and the FTF test are validated tools used in several trials investigating the same combination of substances in the treatment of LBP [17].

### 2.5. Sample Size Calculation

The determination of the sample size for this clinical study was to ensure sufficient power to detect a meaningful difference between the IMPs. A total of 140 subjects were planned for enrollment, to be randomly assigned in a 1:1 ratio, meaning 70 subjects would receive the Test product and 70 subjects would receive the Reference product.

The primary basis for this sample size calculation was the primary endpoint of the study. The study was specifically designed to detect a significant difference of 10 mm that corresponds to the MID in VAS scores [17].

Statistical calculations for the sample size assumed a common standard deviation (SD) of 17 mm based on previous studies [18]. With a targeted statistical power of 90% and a one-sided significance level of 2.5%, an initial minimum of 124 patients were determined to be necessary. To account for an anticipated 10% dropout rate in the primary analysis the total sample size was adjusted upwards to 140 patients, thereby ensuring the maintenance of the predetermined statistical power.

The method for this sample size calculation involved the use of Microsoft EXCEL software (Microsoft Corporation, Redmond, WA, USA) to solve a specific formula that incorporates percentiles of Student’s t distribution, the common standard deviation, and the lower value of difference considered statistically significantly positive. Initially, standard normal distribution percentiles were used, and then an iterative process was applied to converge to the precise number of subjects needed. This EXCEL calculation was further verified for accuracy by employing SAS^®^ statistical software version 9.4 (SAS Institute Inc., Cary, NC, USA) PROC POWER with identical parametrization.

### 2.6. Randomization

Patients in the trial were assigned to treatment groups using a centralized randomization process conducted via an EDC system. A blocked randomization was carried out by a designated third-party principal biostatistician, who generated the randomization scheme prior to the start of the study. The allocation followed a 1:1 ratio between the two treatment groups.

The randomization was performed separately for each study site. A permuted block design with a block size of 2 was applied. This ensured a balanced allocation of treatments across patients within each site.

The randomization scheme was produced using a computerized random-number generator based on the RANUNI function of SAS^®^ statistical software version 9.4. A fixed seed number was generated and stored along with the SAS^®^ program/code to ensure reproducibility of the randomization process.

The clinical trial randomization list was maintained by the independent biostatistician in a secure location with restricted access. Once a subject was randomized and assigned a treatment, the randomization number was not reused or reassigned. Randomization numbers did not overlap with subject numbers.

### 2.7. Statistical Analysis

The statistical and analytical plans for the study were comprehensively described in the study protocol and were pre-specified in the SAP which was finalized prior to the availability of outcome data. The analysis was conducted using SAS^®^ statistical software version 9.4.

The statistical analysis methods employed follow the provisions of ICH E9, where analysis of superiority trials is based on the full analysis set, defined to be as close as possible to including all randomized subjects.

Three population analysis sets were planned for this study: the Per Protocol (PP) population, the Intent-to-Treat (ITT) population, and a safety population. The PP population included randomized subjects who met all inclusion/exclusion criteria, received the assigned IMP, and completed evaluation within the designated visit window without protocol deviations affecting treatment evaluation. The ITT population encompassed all randomized subjects who received an IMP and were to be analyzed according to their initial allocation, irrespective of actual treatment received, withdrawals, or crossovers. Subjects who discontinued prematurely due to lack of treatment effect or worsening condition, requiring alternative therapy, were to be included in the ITT analyses using Last Observation Carried Forward (LOCF) imputation, but not in the PP set. For sensitivity purposes, alternative imputation methods like multiple imputation or modeling approaches were considered depending on the rate and type of missingness. Missing data were to be described by presenting the number and percentage of subjects in the missing category, and all collected data would be used, with no exclusion of participant data sets for missing variables. No subgroup analyses or interim analyses were planned for the study.

For the primary measure of therapeutic superiority, the ITT population was designated as definitive, while results from the PP population would serve as supportive. The primary hypothesis intended to be tested was that the mean difference in VAS changes from baseline between the Test and Reference IMPs would be positive, indicating superiority of the Test product. This hypothesis was planned to be tested using Analysis of Covariance (ANCOVA), with the baseline VAS score included as a covariate and treatment as a factor in the model, along with any baseline characteristics found to be statistically different. A non-parametric rank-based ANOVA would be considered if the data were significantly skewed and the analysis would be performed on the ranks of the change in VAS pain score. Superiority would be established if the mean change for the Test treatment group was statistically significantly greater than the Reference group at a 2.5% significance level (*p* < 0.025), using a one-sided test based on the ITT population with LOCF for missing data.

Similarly to the primary endpoint, the ITT population was intended to be definitive for secondary efficacy measures, with PP population providing supportive results. Hypotheses for these secondary endpoints aimed to show a positive mean difference in changes from baseline for continuous variables (VAS, FTF) or a greater proportion of responders for categorical variables (pain reduction percentage) in the Test product group compared to the Reference. ANCOVA was planned for continuous secondary endpoints, adjusting for baseline scores/distances, while chi-squared or Fisher’s exact tests were designated for categorical secondary endpoints. These comparisons, although using inherently two-sided statistical tests, would interpret rejection of the null hypothesis in a one-sided direction given the observed values, as the comparisons were between two groups.

Regarding safety, no formal safety hypotheses were planned. The safety analysis was intended to focus on a comprehensive descriptive assessment of the frequency and characteristics of Treatment-Emergent Adverse Events (TEAEs) in the safety population, which primarily corresponds to the PP analysis set for safety endpoints. This included summarizing the absolute and relative frequencies of subjects experiencing serious and non-serious topical and systemic AEs, as well as Adverse Drug Reactions (ADRs) and application site reactions. Comparisons of the frequencies of ADRs and application site reactions between the Test and Reference products were planned using chi-squared or Fisher’s exact tests.

A methodological note should be made as ANCOVA, chi-square test and Fisher’s exact test are inherently two-sided tests, but if they reject the null hypothesis the direction of the alternative hypothesis can be one-sided given the observed values, as long as one pays attention to the *p*-value, given that the comparisons are between two groups: test and reference.

## 3. Results

### 3.1. Participant Disposition and Baseline Characteristics

**Figure 1:**
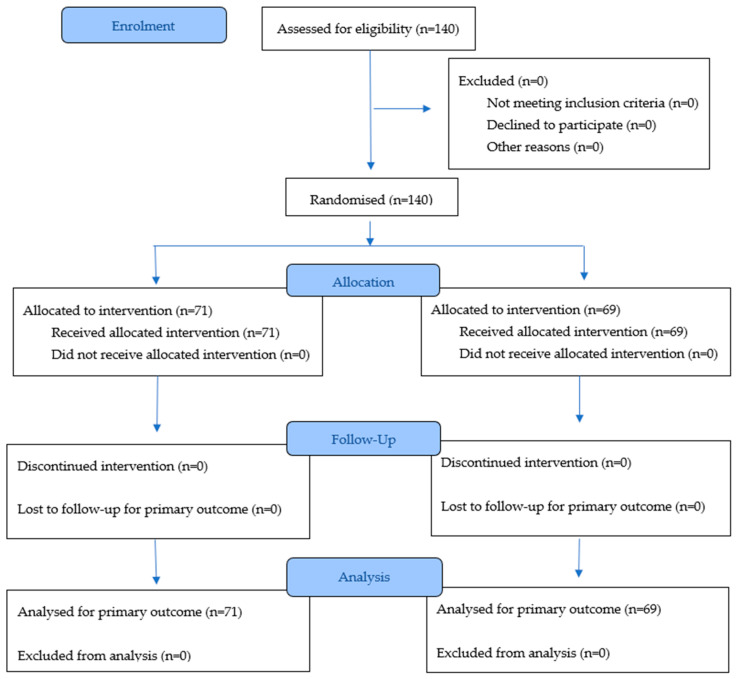
Participant disposition.

Patients were enrolled between September 2024 and June 2025. Following the screening process, a total of 140 subjects were identified as eligible and were subsequently randomized into the study. As per the study’s specific assumption, all 140 randomized patients completed the study in strict accordance with the protocol, and none were lost to follow-up or discontinued prematurely after randomization. There were no post-randomization withdrawals due to adverse events, lack of efficacy, or non-compliance (Figure 1).

No protocol deviations occurred during the conduct of this study. All patients met the inclusion/exclusion criteria, received the assigned treatment, and did not receive any excluded concomitant medications. There were no deviations in patient management or assessment.

Treatment compliance is considered 100% by design, as all IMPs were administered intramuscularly by trained, unblinded study staff. As noted, compliance is assumed due to direct administration by the team, eliminating reliance on patient adherence.

Baseline characteristics (age at baseline and sex) and baseline VAS scores were analyzed (Table 1). From the analysis of the baseline values, it was observed that there were no statistically significant differences between the two treatment groups in age and gender. On the other hand, VAS score was statistically significantly greater in the Diclofenac & Thiocolchicoside group (*p*-value = 0.0194, Wilcoxon two-sample test).

### 3.2. Statistical and Analytical Issues

The statistical analysis was performed in accordance with the SAP. Before any comparative analysis was carried out, the variables under analysis were tested for normality.

Based on normality tests, data were significantly skewed, therefore parametric ANCOVA was avoided. A non-parametric rank based ANCOVA was conducted as more appropriate, and analysis was performed on the ranks of the change in VAS pain scores.

As specified in the SAP, the baseline VAS score was included as a covariate in the analysis. This methodological approach aims to adjust for the initial differences in pain intensity between groups. More importantly, it enables a more accurate assessment of the efficacy of the Test and Reference treatments by accounting for each subject’s baseline pain level, thereby contextualizing the magnitude of pain relief achieved.

In this study, no patients discontinued participation prematurely; therefore, no dropouts occurred. All enrolled subjects completed the study as per protocol, and no early terminations were reported. Consequently, analysis of dropout-related factors such as time to discontinuation, treatment group imbalance, or reason for withdrawal was not applicable. Additionally, no missing data were identified in the final data set. As such, no imputation methods, estimations, or derivations were required. The completeness of data across all participants enhances the reliability of the analyses and eliminates the need for bias adjustment procedures related to incomplete data handling.

Study unblinding did not occur until database lock, after all decisions on data evaluability had been made and documented. There was no intentional or unintentional breaking of the blind identified during the course of the study.

No interim analyses, whether formal or informal, pre-planned or ad hoc, were conducted at any stage during the clinical trial. The study was executed in full compliance with the pre-specified protocol and the approved SAP, both of which did not include provisions for interim data reviews.

Given the limited number of patients enrolled per site, individual site-level analyses lacked sufficient statistical power to yield reliable inferential comparisons. Additionally, no exploratory subgroup analyses were conducted, and none were scheduled to assess the consistency of treatment effects across subgroups (e.g., sex or age group). The total study sample was not powered to permit these exploratory evaluations nor to detect modest interaction effects within subgroups.

### 3.3. Analysis of Efficacy

#### 3.3.1. Primary Endpoint Analysis

The following primary hypotheses were tested:

**H0.**  *The mean difference in the changes from baseline between test and reference IMPs is zero VS*.

**H1.**  *The mean difference in the changes from baseline between test and reference IMPs is positive*.

Rejection of the null hypothesis H0 supports the conclusion of superiority of the Test product against the Reference product.

**Figure 2:**
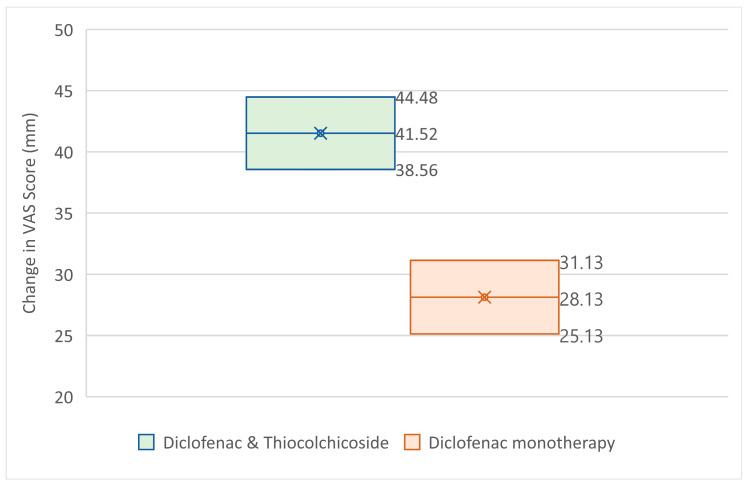
95% CI for VAS change from baseline at Test of Cure Visit.

The ANCOVA was conducted on the change from Baseline to Test of Cure Visit VAS pain score, with treatment as factor in the model and baseline VAS score as co-variate (Table 2). From the analysis (Figure 2), it became clear that the Diclofenac & Thiocolchicoside group had statistically significantly greater improvement in VAS-score (41.52 mm) than the Diclofenac monotherapy group (28.13 mm) with a *p*-value < 0.0001, taking under consideration the baseline values for each group (which did impact the outcome with a *p*-value smaller than 0.0001, without affecting the superiority of the Diclofenac & Thiocolchicoside therapy).

#### 3.3.2. Secondary Efficacy Endpoint Analysis

The following secondary hypotheses were tested, for the mean difference in the change in VAS from baseline to 1 h after administration, and the change in FTF distance from baseline to 1 and 3 h post administration:

**H0.**  *The mean difference in the change from baseline between test and reference IMPs is zero VS*.

**H1.**  *The mean difference in the change from baseline between test and reference IMPs is positive*.

Rejection of the null hypothesis H0 supported the conclusion of superiority of the Test product against the Reference product.

The ANCOVA was conducted on the changes from Baseline to Visit 2 VAS pain score, and the change in FTF distance from baseline to 1 and 3 h post administration, with treatment as factor in the model and the baseline score/distance correspondingly as cofactor (Table 3).

**Figure 3:**
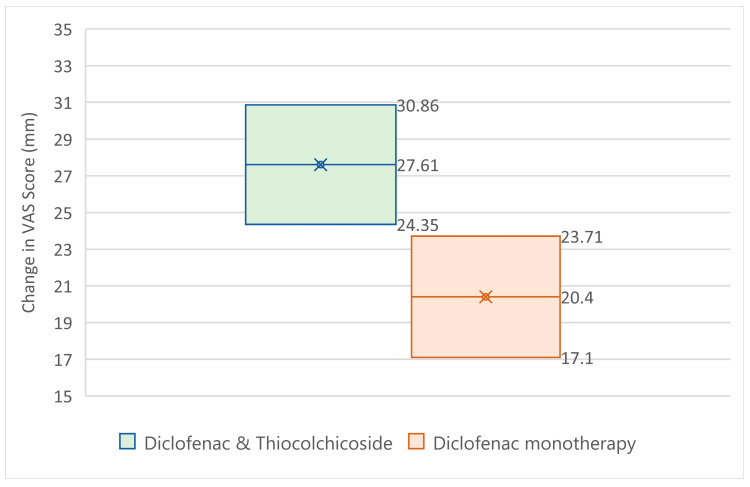
95% CI for VAS change from baseline at visit 2.

From the analysis (Figure 3), it becomes clear that the Diclofenac & Thiocolchicoside group had statistically significantly greater improvement in VAS-score at the 1 h post visit (27.61 mm) than the Diclofenac monotherapy group (20.40 mm) with a *p*-value of 0.0089, taking under consideration the baseline values for each group (which did impact the outcome with a *p*-value smaller than 0.0001, without affecting the superiority of the Diclofenac & Thiocolchicoside therapy).

**Figure 4:**
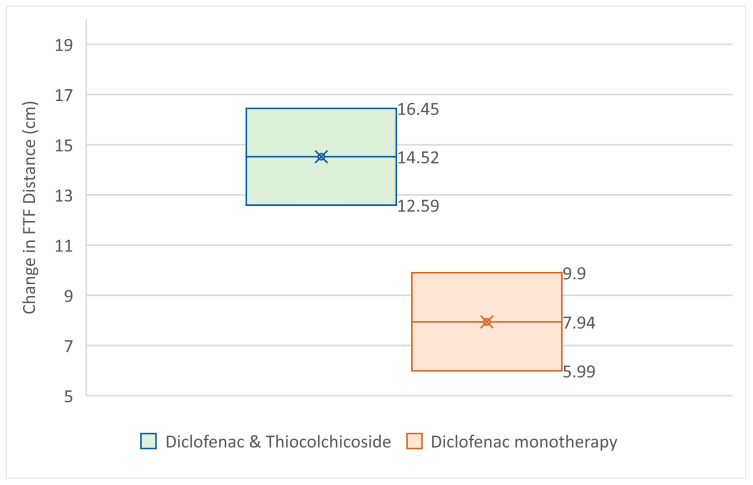
95% CI for FTF distance change from baseline at Test of Cure Visit.

Results for changes from Baseline to the Test of Cure Visit for the finger to floor distance are presented in Table 4 and Figure 4.

From the above analysis it becomes clear that the Diclofenac & Thiocolchicoside group had statistically significantly greater improvement in finger-to-floor distance at the Test of Cure visit (14.52 cm) than the Diclofenac monotherapy group (7.94 cm) with a *p*-value < 0.0001, taking under consideration the baseline values for each group (which did impact the outcome with a *p*-value smaller than 0.0001, without affecting the superiority of the Diclofenac & Thiocolchicoside therapy).

**Figure 5:**
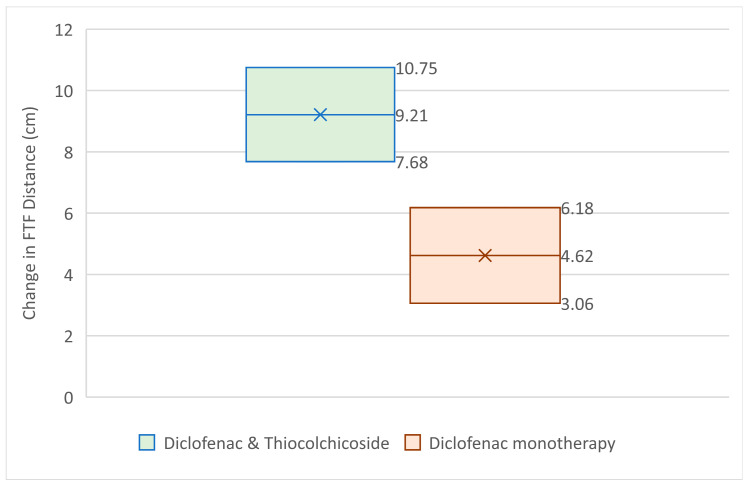
95% CI for FTF distance change from baseline at visit 2.

Results for changes from Baseline to the 1 h post Visit for the finger to floor distance are presented in Table 5 and Figure 5.

From the above analysis, it becomes clear that the Diclofenac & Thiocolchicoside group had statistically significantly greater improvement in finger-to-floor distance at visit 2 (9.21 cm) than the Diclofenac monotherapy group (4.62 cm) with a *p*-value < 0.0001, taking under consideration the baseline values for each group (which did impact the outcome with a *p*-value of 0.0237, without affecting the superiority of the Diclofenac & Thiocolchicoside therapy).

For the proportion of patients with >30% reduction in pain intensity from baseline at 1 and 3 h the following secondary hypotheses were tested:

**H0.**  *The proportion of patients with >30% reduction in pain intensity from baseline is equal between test and reference IMPs*.

**H1.**  *The proportion of patients with >30% reduction in pain intensity from baseline is greater in the test IMP compared to the reference IMP*.

Rejection of the null hypothesis H0 supported the conclusion of superiority of the Test product against the Reference product. The hypotheses were tested using chi-squared test and Fisher’s exact test.

**Figure 6:**
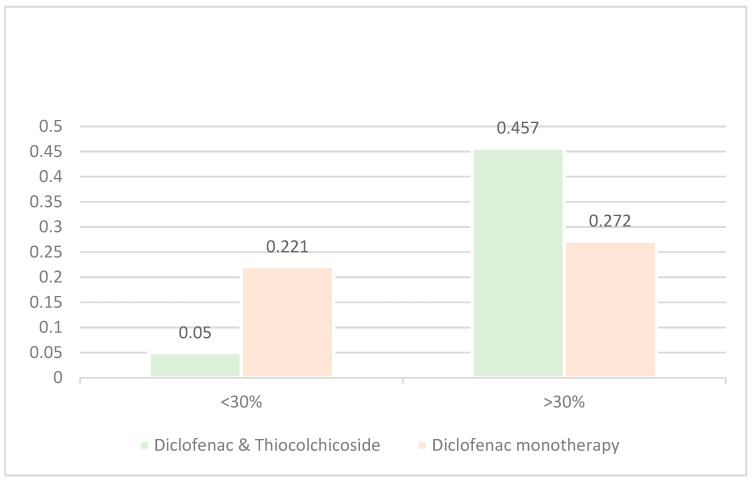
Proportion of patients with >30% reduction in pain intensity at Test of Cure visit.

The results for 3 h post treatment administration are presented in Table 6 and Figure 6.

The above results support the main analysis as the Diclofenac & Thiocolchicoside group has a statistically significantly greater proportion of patients experiencing a >30% reduction in pain intensity (45.7%) than the Diclofenac monotherapy group (27.2%) with a *p*-value < 0.0001.

**Figure 7:**
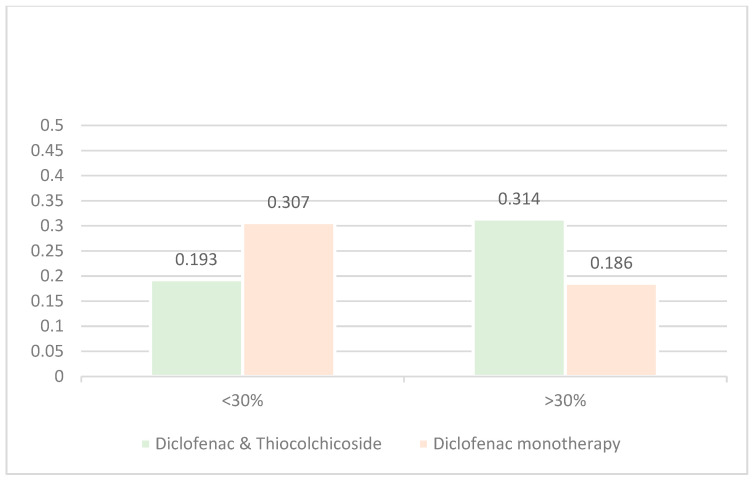
Proportion of patients with >30% reduction in pain intensity at visit 2.

The results for 1 h post treatment administration are presented in Table 7 and Figure 7.

The above results support the main analysis as the Diclofenac & Thiocolchicoside group has a statistically significantly greater proportion of patients experiencing a >30% reduction in pain intensity (31.4%) than the Diclofenac monotherapy group (18.6%) with a *p*-value of 0.0066 (Fisher’s exact test).

#### 3.3.3. Safety Evaluation

In this clinical study, the overall incidence of AEs was low. A total of one AE was reported, occurring in the FDC arm. No adverse events were reported in the Diclofenac monotherapy arm (Table 8).

The single AE reported was a mild headache experienced by a 74-year-old female participant approximately one hour after administration of the IMP. The headache resolved without intervention by the time of the three-hour post-dose assessment. Notably, the participant had concurrently received concomitant medication containing felodipine for hypertension. Headache is a known adverse reaction associated with all active substances in IMPs and concomitant medication. Therefore, the event was assessed as possible in terms of causality.

The event was assessed as mild in intensity, non-serious, and transient. It was coded using MedDRA version 28.0 as Headache (MedDRA code: 10019211), under the Nervous System Disorders System Organ Class (SOC code: 10029205). No other adverse events or safety concerns were observed in either study arm.

This isolated event did not necessitate any modification to study procedures and had no impact on the participant’s continuation in the study.

## 4. Discussion

The primary efficacy analysis, based on the ITT population, demonstrated statistically significant superiority of the Test treatment over the Reference treatment in reducing pain, as measured by the change in VAS score from baseline to 3 h post administration. The observed treatment difference supports the conclusion of therapeutic superiority.

Secondary efficacy endpoints, including 1 h VAS change, FTF scores at 1 and 3 h post administration, and the responder analysis, also showed statistically significant advantages for the Test treatment, reinforcing the primary efficacy findings.

Taken together, these findings provide strong and internally consistent evidence of the superior efficacy of the combination of Diclofenac and Thiocolchicoside FDC product compared to Diclofenac monotherapy treatment in the management of acute pain under the conditions of this study. The ~13 mm greater pain reduction with combination exceeds the 10 mm MID for VAS in acute pain, indicating a clinically meaningful benefit. Additionally, nearly half of combination-treated patients achieved >30% pain relief vs. about a quarter with monotherapy.

The IMPs were well tolerated in this study. Only one adverse event, a mild and transient headache, was reported in a single participant in the FDC group. No serious adverse events, events requiring withdrawal from treatment, or initiation of additional concomitant medications occurred.

The safety profile observed in this trial supports the continued clinical development and potential therapeutic use of the investigational product.

Overall, the results of the study agree with those of recent systematic findings on the combination of Diclofenac and Thiocolchicoside [17], demonstrating superiority in pain relief and mobility over Diclofenac monotherapy, higher than the established MID.

Prior trials evaluating this combination exhibited moderate to high risk of bias [17], particularly concerning blinding, randomization, statistical analysis, and pain intensity thresholds. For instance, Iliopoulos et al. [18] was single-blind, and Akhter & Siddiq [12] was open-label. In contrast, this randomized, double-blind, active-controlled trial improves upon prior designs by incorporating more rigorous blinding, pre-specified analyses, and refined eligibility criteria. These enhancements strengthen the reliability of the conclusions.

Internal validity was strong: centralized randomization and stringent blinding achieved through identical secondary packaging and the use of dedicated unblinded personnel for drug preparation and administration minimized selection, performance, and detection bias. Follow-up was complete (no dropouts or missing data), eliminating attrition bias and the need for imputation, and the study adhered strictly to the pre-specified protocol and analysis plan. The trial was adequately powered (n = 140; 90% power) to detect a meaningful 10 mm difference on the VAS. Endpoints were clinically relevant and complementary; VAS pain intensity as a gold-standard patient-reported measure, finger-to-floor distance as an objective functional index, and a ≥30% pain-reduction responder analysis to anchor results to a pragmatic threshold of benefit. Importantly, the chosen primary endpoint is one commonly employed in trials of the Diclofenac-Thiocolchicoside combination, enabling direct comparison with prior studies and strengthening external interpretability. Statistical methods were appropriate and prespecified, using ANCOVA to adjust for baseline pain. Additional bias-control measures included an ethically appropriate active comparator while maintaining blinding, direct observation of dosing to ensure full compliance, and the absence of interim looks that could inflate type-I error. External validity is supported by the multicenter conduct and broadly representative inclusion of adults with acute non-specific LBP of moderate-to-severe intensity, and the short-term safety profile was reassuring, with no serious adverse events.

Several limitations warrant consideration. First, the absence of a placebo arm precludes quantification of absolute placebo effects and the isolated contribution of thiocolchicoside; however, this was ethically and pragmatically mitigated by using an active control consistent with acute pain care, and blinding/packaging strategies minimized expectancy bias. Second, generalisability is constrained by necessary exclusions (e.g., serious pathologies, significant comorbidities, pregnancy/lactation), so findings apply chiefly to otherwise healthy adults with acute non-specific LBP; this was partly mitigated by the multicenter setting and broad age range, which enhance applicability to common urgent-care contexts. Third, the observation window was intentionally short (efficacy to 3 h, safety to 24 h), limiting inferences about sustained benefit, functional recovery, healthcare utilization, or recurrence; this choice was methodologically justified to capture peak early analgesia, reduce confounding from subsequent care, and minimize exposure to thiocolchicoside, but longer-term outcomes remain beyond the study’s scope. Additionally, a baseline imbalance in VAS scores was noticed between the two treatment arms, although it was adjusted for in the analysis. Finally, no subgroup analyses were performed; while this avoids multiplicity and underpowered interaction testing, it limits conclusions about differential effects across baseline severity, age, or sex. This was mitigated analytically by covariate adjustment for baseline pain and by balanced demographics at entry. Taken together, these limitations were anticipated and addressed where feasible; importantly, none undermine the central inference of superior short-term analgesia and early functional improvement with the FDC compared with diclofenac alone, while highlighting priorities for future research on durability of effect and broader populations.

Taken together, the results support considering the combination of Diclofenac and Thiocolchicoside as a pragmatic early-management option for appropriately selected adults with acute non-specific LBP, where rapid pain relief and recovery of mobility are priorities. Although longer-term data are needed, the consistent superiority across pain and function, coupled with favourable short-term safety, strengthens the rationale for targeted combination therapy in routine practice.

## Figures and Tables

**Table 1:** Demographics and Baseline Characteristics by Treatment Group.

Baseline Characteristics	Test Group (n = 71)	Reference Group (n = 69)
Sex	Female: n = 38 (53.5%)	Female: n = 31 (44.9%)
	Male: n = 33 (46.5%)	Male: n = 38 (55.1%)
Age	49 ± 15	48 ± 15
Muscle Spasm	Yes: n = 63 (88.7%)	Yes: n = 54 (78.3%)
	No: n = 8 (11.3%)	No: n = 15 (21.7%)
VAS Score	78.39 ± 10.92	73.52 ± 11.29
FTF Score	34.04 ± 11.19	30.83 ± 11.77

**Table 2:** ANCOVA table for VAS change from baseline at Test of Cure Visit.

Source	df	Type III SS	Mean Square	F Value	*p*-Value
Treatment	1	35,191.7	35,191.7	39.23	<0.0001
Baseline VAS-score	1	46,248.3	46,248.3	51.55	<0.0001
**Therapy group**			**Estimated mean change in VAS-score**		
Diclofenac monotherapy			28.13		
Diclofenac & Thiocolchicoside			41.52		

**Table 3:** ANCOVA table for the VAS change from baseline at visit 2.

Source	df	Type III SS	Mean Square	F Value	*p*-Value
Treatment	1	9264.03	9264.03	7.04	0.0089
Baseline VAS-score	1	26,419.60	26,419.60	20.09	<0.0001
**Therapy group**			**Estimated mean change in VAS-score**		
Diclofenac monotherapy			20.40		
Diclofenac & Thiocolchicoside			27.61		

**Table 4:** ANCOVA table for the FTF distance change from baseline at Test of Cure visit.

Source	df	Type III SS		Mean Square	F Value	*p*-Value
Treatment	1	30,182.4		30,182.4	24.46	<0.0001
Baseline FTF distance	1	20,215.4		20,215.4	16.38	<0.0001
**Therapy group**			**Estimated mean change in finger-to-floor distance**			
Diclofenac monotherapy			7.94			
Diclofenac & Thiocolchicoside			14.52			

**Table 5:** ANCOVA table for the FTF distance change at visit 2.

Source	df	Type III SS		Mean Square	F Value	*p*-Value
Treatment	1	23,548.4		23,548.4	16.89	<0.0001
Baseline FTF distance	1	7293.8		7293.8	5.23	0.0237
**Therapy group**			**Estimated mean change in finger-to-floor distance**			
Diclofenac (monotherapy)			4.62			
Diclofenac & Thiocolchicoside			9.21			

**Table 6:** Comparison of proportion of patients with >30% reduction in pain intensity at Test of Cure visit.

Proportion of Patients with >30% Reduction in Pain Intensity by Treatment				
Treatment Group				
		Diclofenac Monotherapy	Diclofenac & Thiocolchicoside	Total
≤30%	N	31	7	38
	%	22.1	5.0	27.1
>30%	N	38	64	102
	%	27.2	45.7	72.9
Total	N	69	71	140
	%	49.3	50.7	100.0
*p*-value	Chi-squared	<0.0001	Fisher’s Exact	<0.0001

**Table 7:** Comparison of proportion of patients with >30% reduction in pain intensity (visit 2).

Proportion of Patients with >30% Reduction in Pain Intensity by Treatment				
Treatment Group				
		Diclofenac Monotherapy	Diclofenac & Thiocolchicoside	Total
≤30%	N	43	27	70
	%	30.7	19.3	50.0
>30%	N	26	44	70
	%	18.6	31.4	50.0
Total	N	69	71	140
	%	49.3	50.7	100.0
*p*-value	Chi-squared	0.0041	Fisher’s Exact	0.0066

**Table 8:** Display of AE frequency per group.

Adverse Events		Treatment		Total
		Test Group	Reference Group	
No	N	70	69	139
	%	98.6	100.0	99.3
Yes	N	1	0	1
	%	1.4	0.0	0.7
Total	N	71	69	140
	%	100.0	100.0	100.0

## Data Availability

The data supporting this study are subject to legal/regulatory restrictions. In line with Regulation (EU) No. 536/2014, the clinical study report will be submitted to CTIS after marketing authorisation is granted; the public version will thereafter be available via CTIS. Data will not be shared prior to the authorisation decision.

## Supplementary Materials

The following supporting information can be downloaded at: https://www.mdpi.com/article/10.3390/jcm14248827/s1, Table S1: CONSORT 2025 checklist item description.

## Abbreviations

The following abbreviations are used in this manuscript:

AE	Adverse Events
ADR	Adverse Drug Reaction
ANCOVA	Analysis of Covariance
CTIS	Clinical Trials Information System
EDC	Electronic Data Capture
EMA	European Medicines Agency
EOF	Greek National Organization for Medicines
FDC	Fixed-Dose Combination
FTF	Finger-to-Floor
GCP	Good Clinical Practice
IM	Intramuscular
IMP	Investigational Medicinal Product
ITT	Intent-to-Treat
LBP	Low Back Pain
LOCF	Last Observation Carried Forward
NSAID	Non-steroidal Anti-inflammatory Drug
PP	Per Protocol
SAP	Statistical Analysis Plan
SD	Standard Deviation
TEAE	Treatment-Emergent Adverse Event
VAS	Visual Analogue Scale
YLD	Years Lived with Disability
